# Regular exposure to non-burning ultraviolet radiation reduces signs of non-alcoholic fatty liver disease in mature adult mice fed a high fat diet: results of a pilot study

**DOI:** 10.1186/s13104-019-4112-8

**Published:** 2019-02-11

**Authors:** Samantha Teng, Lipi Chakravorty, Naomi Fleury, Shelley Gorman

**Affiliations:** Telethon Kids Institute, University of Western Australia, West Perth, PO Box 855, Perth, WA 6872 Australia

**Keywords:** Ultraviolet radiation, Mice, Non-alcoholic fatty liver disease, Obesity, Age, High fat diet

## Abstract

**Objective:**

Obesity often emerges in middle age, increasing risk for metabolic disorders. Our previous preclinical experiments identified that chronic exposure to non-burning ultraviolet radiation, like that achieved through sun exposure, prevented weight gain and signs of metabolic dysfunction in young adult mice fed a high fat diet. Our objective was to perform a pilot study to estimate the effect size of ongoing exposure to sub-erythemal (non-burning, low dose) UVB (1 kJ/m^2^) radiation on measures of adiposity, food intake and physical activity in ‘mature’ adult C57Bl/6J male mice fed a high fat diet for 12 weeks.

**Results:**

The severity of liver steatosis, fibrosis and inflammation were reduced in older adult mice exposed twice a week to ultraviolet radiation (from 29 weeks of age), compared to mock-irradiated mice, with some evidence for reduced hepatic mRNAs for *tnf* and *tgfß1* (not *fatp2* nor *fasN*). Power analyses suggested that up to 24 mice per treatment would be required in future experiments to detect a significant effect on some markers of adiposity such as body weight gain. Our studies suggest frequent exposure to low levels of sunlight may reduce the severity of hepatic steatosis induced in older adults living in environments of high caloric intake.

**Electronic supplementary material:**

The online version of this article (10.1186/s13104-019-4112-8) contains supplementary material, which is available to authorized users.

## Introduction

With aging populations [1] and rising rates of obesity in many countries around the world [2], many people may experience metabolic diseases associated with obesity and aging. In addition to excessive caloric intake and insufficient activity, there may be multiple drivers of obesity and metabolic dysfunction. We are interested in the potential for low dose (sub-erythemal or non-burning) ultraviolet radiation (UVR), like that derived from sun exposure, to affect the development of metabolic dysfunction [3]. We previously observed that long-term exposure to low dose UVB radiation (1 kJ/m^2^, given twice-a-week) reduced weight gain and signs of type-2 diabetes (e.g. reduced blood glucose measured during a glucose tolerance test) and non-alcoholic fatty liver disease (NAFLD) in young adult C57Bl/6J male mice fed a high fat diet (from 8 weeks of age) [4, 5]. Our studies suggested that some of these effects (e.g. reduced liver steatosis) were dependent on the release of nitric oxide from UV-irradiated skin [4, 5].

Aging affects dermal responses to UVR exposure. For example, skin from older people (77–82 years-old) has significantly reduced capacity to produce vitamin D_3_ when exposed to UVR [6]. Similarly, body composition changes with age, and increases in fat mass and declines in fat-free mass are accompanied by metabolic changes [7]. Mice develop a more severe metabolic phenotype when high fat diet feeding commences in older age [8]. Therefore, the effects of UVR exposure in older mice fed a high fat diet may be different to younger animals. Here, we report findings of a pilot study done in older ‘mature’ adult C57Bl/6J male mice (from 29 weeks of age) fed a high fat diet, completed to estimate the effect size (if any) of UVR exposure upon highly variable adiposity outcomes, with the goal of using this information to adequately power future studies. We also compared the effects of UVR on signs of liver inflammation in these older mice to young adult mice using archived specimens from our published studies [4, 5].

## Main text

### Materials and methods

#### Mice

This study was approved by the Telethon Kids Institute Animal Ethics committee (AEC#238) and was carried out in accordance with the ethical guidelines of the National Health and Medical Council (Australia). Naïve C57Bl/6J male mice were obtained from the Animal Resources Centre (Murdoch, Western Australia). Mice were housed in specific-pathogen-free conditions and kept individually (open-topped cages, aspen chip bedding) with temperature controlled (21 ± 1 °C, mean ± range), Perspex-filtered fluorescent lighting (12-h light/dark cycle) and unlimited access to food and water. One mouse from the UVR-irradiated treatment group was euthanized due to a dermatitis that did not resolve, with all data for this mouse excluded from the dataset. The experiment was conducted between August and December 2015.

#### Diets

**Fig. 1:**
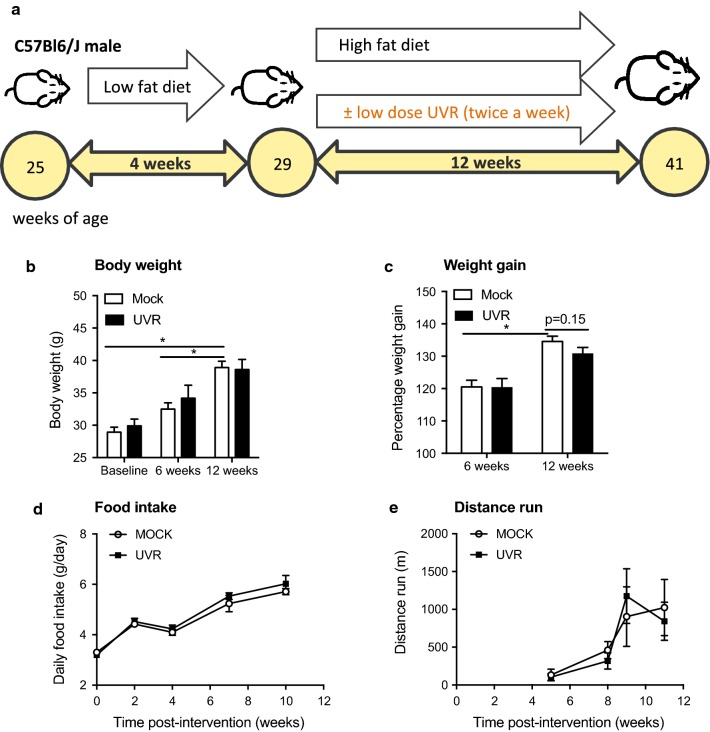
Regular exposure to UVR did not modify body weight, weight gain, food intake or distance run by mature adult mice fed a high fat diet. Fourteen 25 week-old C57Bl/6J male mice were fed a vitamin D-deficient low fat diet for 4 weeks. From 29 weeks of age, mice were fed a high fat diet for a further 12 weeks. The shaved dorsal skin of these mice were treated twice a week with either: (1) sub-erythemal UVR (1 kJ/m^2^ UVB); or, (2) were mock irradiated (Mock UVR). Mice were treated for 12 weeks with the mock or UVR treatments and fed a high fat diet until 41 weeks of age. There were 7 mice per treatment. nb. One mouse was euthanized from the UVR treatment due to the development of a severe dermatitis, which did not resolve. In **a**, is an overview of the experiment. In **b**, body weights at baseline and after 6 and 12 weeks, and, **c** body weight gain (as a % of baseline) after 6 and 12 weeks of being fed the high fat diet. In **d**, is food intake per day. In **e**, distance run was measured for the first 3 h immediately following a mock or UVR treatments after 5, 8, 9 and 11 weeks of feeding mice the high fat diet. Data is shown for n = 7 mock-irradiated and n = 6 UVR-exposed mice in **b**–**d**, and n = 4/treatment in **e**. All values are expressed as the mean +/± SEM, with (*) denoting significant differences between time points (p < 0.05)

Mice were fed a low fat diet containing 5.0% fat (not fortified with vitamin D_3_) for 4 weeks from 25 weeks of age (25 ± 2 weeks, mean ± SEM, Fig. 1a) as diets fortified with vitamin D_3_ can interfere with the suppressive effects of exposure to UVR [5]. Mice were then fed a high fat diet containing 23.9% fat (not fortified with vitamin D_3_) for a further 12 weeks. For diet composition (Specialty Feeds, Glen Forrest, Western Australia) see Additional file 1: Table S1.

#### UVR treatment

Mice were exposed twice a week (Monday and Friday mornings) to either 1 kJ/m^2^ (sub-erythemal, non-burning) UVB radiation (UVR; n = 7) or no UVR (Mock; n = 7) for 12 weeks of high fat diet feeding (Fig. 1a) after random allocation into treatment groups. UVR was administered as previously described from six 40 W lamps (TL UV-B; Philips, Eindhoven, the Netherlands) emitting broad spectrum UVR (250–360 nm), of which 65% was UVB radiation (280–315 nm), exposing shaven dorsal skin to 1 kJ/m^2^ (suberythemal) UVB radiation. UVC radiation (< 280 nm) was blocked using 0.2 mm PVC plastic [9]. During treatment mice were individually housed in a Perspex container. Mock-irradiated mice were then exposed to non-UVR emitting fluorescent lighting and otherwise handled in the same fashion as mice exposed to UVR. Dorsal fur of all mice was removed prior to the first treatment with UVR and then every 2 weeks by clippers.

#### Measuring weight gain and food intake

Mice were weighed weekly using a digital scale (0.1 g sensitivity; Scout) every Friday morning in a random fashion. To determine food intake, the amount of diet present in food hoppers was determined using the digital scale, with the mean daily food intake calculated for 4 × 24 h periods (Monday to Friday) [10].

#### Determining adipose tissue mass by dissection of tissue

At the conclusion of the experiment, gonadal white adipose tissue (gWAT) and interscapular brown adipose tissue (BAT) was dissected and weighed (0.0001 g sensitivity, Analytical Standard Electric Balance) (3–4 mice/treatment, mock then UVR treated mice).

#### Determining adipose tissue volume by MRI

Following humane euthanasia (anaesthetic overload with isofluorane inhalation, done to limit damage to internal structures), the corpses of 3 mice per treatment were frozen at − 80 °C, and thawed prior to imaging using a preclinical MRI (MRS 3000™ Series scanner) at the ACRF Cancer Imaging Facility (Harry Perkins Institute for Medical Research, Perth, Western Australia). OsiriX Lite Imaging Software (v8.0.1: Geneva, Switzerland) was used to quantify the BAT, visceral WAT (vWAT), and subcutaneous WAT (scWAT) from MRI transversely sliced at 0.1 mm thickness (28 slices, mock then UVR treated mice). BAT was identified by a characteristic hyperintensity in T1-weighted images in the interscapular fat within the cervical and upper thoracic segments [11]. scWAT and vWAT in thoracic and abdominal cavities (neck to tail) were identified as light grey areas superficial and deep to the thoracic or abdominal walls, respectively. To determine volume, the sum of all areas of BAT, vWAT (abdominal and thoracic) and scWAT (abdominal and thoracic) for each transverse cross sectional slice (n = 28) was calculated and multiplied by slice thickness (0.1 mm) [11].

#### Measuring voluntary wheel running

In weeks 5, 8, 9 and 11 of being fed the high fat diet, 4 mice per group were individually housed in a new cage installed with a wireless running wheel (Med Associates Inc., Vermont, USA) immediately after exposure to UVR/mock treatment. Mice had unrestricted access to running wheels until their removal with distance run measured for up to 3 days, with the number of rotations from each running wheel was recorded every 30 s (Wheel Manager Software, Med Associates Inc).

#### Histopathological assessment for signs of NAFLD

NAFLD severity was assessed by blinded-scoring of formalin-fixed, H&E- and Masson’s trichrome-stained liver sections for the degree of steatosis (combined steatosis and hepatocellular ballooning, H&E) and fibrosis (Masson’s) using the non-alcoholic steatohepatitis (NASH) scoring system as previously described [4, 12]. Steatosis (≤ 6) was combined with fibrosis (≤ 4) scores to produce a total score of ≤ 10 (3–4 mice/treatment). Inflammatory foci/field were identified as tight bundles of eosinophilic-staining and multinucleated cells, with the mean number per field (at 20× magnification, H&E) counted in a blinded fashion for 5 fields per mouse (3–4/treatment).

#### Plasma metabolites

Plasma levels of alanine aminotransferase (ALT), aspartate aminotransferase (AST), glucose, triglyceride, and cholesterols (HDL-, (measured) LDL-) were measured at PathWest Pathology, using the Clinical Chemistry kit as part of the Architect *c* System (Abbot Laboratories, Weisbanden, Germany) for 3–4 mice/treatment.

#### Detection of mRNA

At the end of the experiment, mRNA was extracted from snap-frozen livers of mice (n = 3–4/treatment) with cDNA synthesized and real-time assays performed as previously described [13, 14] using Quantitect Primer Assays (Qiagen, Doncaster, VIC, Australia) for detection of *tnf, tgfß1, fatp2* and *fasN*, with internal primers used for detection of *eef1α,* the house-keeping gene [14]. We also measured mRNA levels in snap-frozen liver specimens retained from published experiments [5], in which young adult mice (treated from 8 weeks of age, n = 14–15/treatment) were fed the low or high fat diet (Additional file 1: Table S1), and exposed (or mock treated) twice a week to 1 kJ/m^2^ UVB radiation for 12 weeks as described in Fig. 1a.

### Statistical analyses

GraphPad Prism (v7.0a for MAC OS10) was used to compare data (unit = single animal) between treatments by unpaired student *t*-tests or one-way ANOVA (with Tukey’s post hoc) or Mann–Whitney or Kruskal–Wallace (with Dunn’s multiple comparison post hoc) tests, depending on whether data was distributed normally, or not (respectively). To compare the effects of time on body weight and weight gain, a two-way ANOVA (with Tukey’s post hoc) was used. The G*Power (v3.1.3 for MAC OS10) program was used to predict the sample size needed for statistically powered experiments, using a two-tailed student’s *t* test (difference between two independent means, a priori analysis at a power of (1 − ß error) probability of 0.8, and α error probability of 0.05). Results are expressed as the mean +/± SEM (unless otherwise stated) and considered statistically significant for *p*-values < 0.05.

### Results

**Fig. 2:**
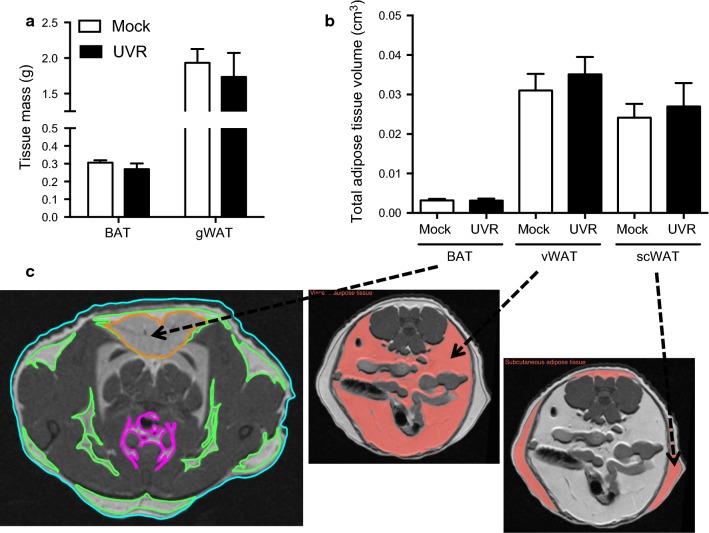
Regular exposure to UVR did not modify BAT or WAT mass or volume in mature adult mice. In **a**, interscapular BAT (BAT) and gonadal WAT (gWAT) mass are shown, dissected from mock- (n = 4) and UVR- (n = 3) irradiated mice at the end of the experiment. In **b**, the volume of BAT, visceral WAT (vWAT) and subcutaneous WAT (scWAT) was determined using MRI (n = 3/treatment). All values in **a** and **b** are expressed as mean + SEM. In **c**, representative transverse MRI images from a mouse are shown, with shading used to identify the different deposits of adipose tissue, from scans performed on euthanized animals at the end of the experiment. Using the OsiriX Lite Imaging Software, areas of BAT (orange), vWAT (purple) and scWAT (green) deposits were identified across 24 × 0.1 mm^2^ sections from the neck to tail. Sections identifying BAT was obtained across the transverse plane of the interscapular region. Examples for vWAT and scWAT are also highlighted (in orange) for a transverse section across the lower abdomen

Increased body weight and weight gain was observed for older mice receiving either UVR or mock treatments with time (Fig. 1b, c). However, ongoing exposure to low dose UVR did not significantly modify body weight (Fig. 1b) or weight gain (compared to baseline, Fig. 1c) compared to mock treatment, after 6 and 12 weeks of eating the high fat diet. There was no effect of exposure to low dose UVR on BAT or gWAT mass weighed at the end of the experiment (Fig. 2a). Similarly, exposure to UVR did not modify the volumes of BAT, vWAT or scWAT (Fig. 2b) measured using MRI (Fig. 2c). There was no effect of ongoing exposure to low dose UVR on daily food intake (Fig. 1d) or distance run (Fig. 1e).

**Fig. 3:**
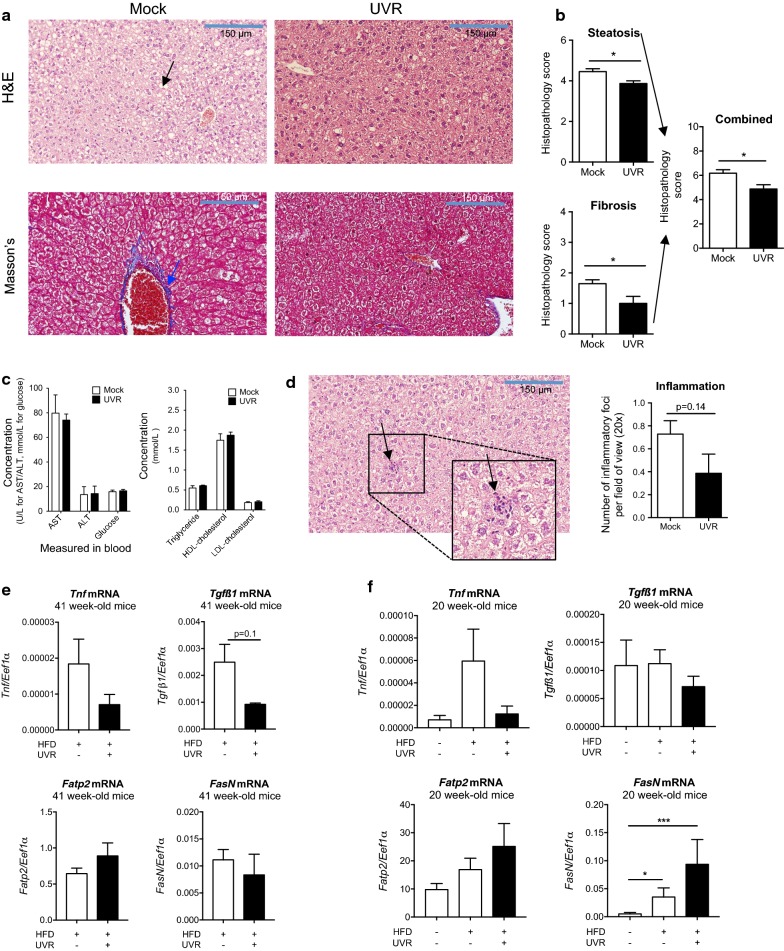
Regular exposure to UVR suppressed the development of signs of NAFLD in mature adult mice. In **a**, representative liver sections from mock- (n = 4) or UVR- (n = 3) treated mice are shown, which were stained with H&E or Masson’s Trichrome, with a black arrow identifying steatosis (H&E), and a blue arrow indicating fibrosis (collagen stained blue, Masson’s) around a blood vessel (×20 magnification). Livers were dissected from euthanised mice at end of the experiment. In **b**, histopathological grading scores are shown were for the extent of steatosis (/6), fibrosis (/4) and both combined (/10). In **c**, plasma levels of ALT, AST, glucose, triglyceride, LDL- and HDL-cholesterol measured at the end of the experiment. In **d**, the number of inflammatory foci (identified by the presence of tight bundles of eosinophilic-staining and multi-nucleated cells, black arrow) were counted per field (×20 magnification) and averaged per mouse liver section stained with H&E. In **e**, *tnf, tgfß1, fatp2* and *fasN* mRNAs were measured in the livers of these mature mice (41 weeks old at the end of the study) with *eef1α* used as the housekeeping control. In **f**, *tnf, tgfß1, fatp2* and *fasN* mRNAs were measured in the livers of 20 week-old mice previously fed a high fat diet (HFD) or low fat diet for 12 weeks (from 8 weeks of age) with some exposed to low dose UVR and all other mice ‘mock-treated’, twice a week throughout this feeding period (n = 14–15/treatment). All values are expressed as the mean + SEM, with (*) denoting a significant difference between treatments (*p < 0.05, ***p = 0.0001)

The severity of liver steatosis and fibrosis were significantly reduced by ongoing exposure to UVR (Fig. 3a, b). No difference was observed in plasma levels of liver enzymes (ALT, AST), glucose, triglyceride or cholesterol (HDL-, LDL-) from mock- or UVR-irradiated mice (Fig. 3c). There was a non-significant reduction in the number of inflammatory foci in mice exposed to UVR, compared to mock-treated mice (Fig. 3d). We also detected non-significant reductions in hepatic levels of *tnf* and *tgfß1* mRNA in both older (Fig. 3e) and younger (Fig. 3f; 20 weeks of age at study end) [5] adult mice exposed to low dose UVR, with *tnf* mRNA less frequently detected in the livers of the younger (26/44, 59%) compared to older (7/7, 100%) mice. Levels of *tgfß1* mRNA were ≥ tenfold more in the livers of the older (Fig. 3e), compared to younger (Fig. 3f) mice. To explore potential mechanisms by which exposure to UVR reduced liver steatosis, mRNA levels of *fatp2* (*fatty acid transport protein 2*) and *fasN* (*fatty acid synthase*), genes central to the regulation of fatty acid transport [15] and de novo lipogenesis [16] (respectively), were assessed. Exposure to UVR did not significantly modify *fatp2* or *fasN* mRNA concentrations in the livers of the older (Fig. 3e) or younger (Fig. 3f) mice, although increased *fasN* mRNA was observed in the livers of the younger mice fed a high fat diet compared to those fed a low fat diet (as previously observed in similar studies [17]).

Sample sizes of 5–24 mice/treatment would be required for adequate power to observe significant statistical differences between mock-irradiated and UVR-exposed mature adult mice for outcomes such as body weight gain, BAT weight, liver histopathology and inflammatory foci and, hepatic *tnf* mRNA levels; with larger sample sizes needed for other outcomes (Additional file 1: Table S2).

### Discussion

This pilot study was conducted to estimate the effect size of UVR exposure upon signs of adiposity and NAFLD in mature adult mice fed a high fat diet. Our power analyses suggested that sample sizes of up to 24 mice/treatment might be required to observe significant effects of low dose UVR on some outcomes, which could be feasible to achieve in terms of animal management and cost. However, it would more difficult to fully power experiments for outcomes requiring ≥ 99 mice/treatment (e.g. gWAT weights). Even with only a small number of mice per treatment, we observed significant effects of ongoing exposure to low dose UVR in reducing signs of NAFLD in the mature mice fed a high fat diet, including reduced hepatic steatosis and fibrosis, and non-significant reductions in inflammatory foci and levels of *tnf* (pro-inflammatory cytokine) and *tgfß1* (pro-fibrotic cytokine) mRNAs, which both promote the development of NASH [18]. These results are consistent with our earlier findings [4, 5] and those of other groups [19]. Reduced signs of liver steatosis were not linked with reductions in the expression of mRNAs of genes governing free fatty acid uptake *(fatp2*) or de novo lipogenesis (*fasN*), with further studies needed to identify mechanism(s) by which exposure to UVR regulates lipid accumulation in the liver, such as through enhanced free fatty acid ß-oxidation [16]. It is likely that increasing age contributed significantly towards the induction of fibrosis and inflammation, as older mice can exhibit increased hepatic macrophage infiltration, serum liver enzymes, and liver mRNA levels of *tgfß1*, *tnf* and other pro-fibrotic and -inflammatory genes in mice fed a high fat diet [20]. Importantly, for this investigation we focused on sub-erythemal (non-burning) doses of UVR, which present as low risk for negative health outcomes otherwise associated with UVR exposure (e.g. skin cancer). Further fully powered experiments are needed to demonstrate how these benefits might occur through more comprehensive quantification of hepatic steatosis (e.g. through Oil-red O staining, measuring hepatic triglyceride) and by examining the effects of low dose UVR on other measures of adiposity and glucose dysfunction (e.g. glucose tolerance tests) in mature and older mice with diet-induced obesity, with an eye towards translational studies in the future in humans.

## Limitations

A number of limitations of this pilot study were identified and included that:

- It was not sufficiently powered for all outcomes;
- There was no control group (e.g. mice fed a low fat diet);
- Mice may not be ideal animal models for studies of the effects of UVR exposure (e.g. nocturnal, hairy);
- Mice were housed individually for measurement of food intake and distance run; and,
- Distance run was not measured in fully ‘trained’ mice.

## Additional file

**Additional file 1: Table S1.** Ingredient list of low fat diet (LFD) and high fat diet (HFD) fed to mice. **Table S2.** Power analysis to determine sample sizes of treatment groups to investigate the effects of exposure to low dose UVR on metabolic outcomes in mature ‘older’ mice fed a high fat diet.

## Abbreviations

ALT: alanine aminotransferase
AST: aspartate aminotransferase
BAT: brown adipose tissue
NAFLD: non-alcoholic fatty liver disease
NASH: non-alcoholic steatohepatitis
UVR: ultraviolet radiation
WAT: white adipose tissue

## References

[1] Lutz W, Sanderson W, Scherbov S (2008). The coming acceleration of global population ageing. Nature.

[2] Collaboration NCDRF (1975). Trends in adult body-mass index in 200 countries from to 2014: a pooled analysis of 1698 population-based measurement studies with 192 million participants. Lancet.

[3] Gorman S, Lucas RM, Allen-Hall A, Fleury N, Feelisch M (2017). Ultraviolet radiation, vitamin D and the development of obesity, metabolic syndrome and type-2 diabetes. Photochem Photobiol Sci.

[4] Fleury N, Feelisch M, Hart PH, Weller RB, Smoothy J, Matthews VB, Gorman S (2017). Sub-erythemal ultraviolet radiation reduces metabolic dysfunction in already overweight mice. J Endocrinol.

[5] Geldenhuys S, Hart PH, Endersby R, Jacoby P, Feelisch M, Weller RB, Matthews V, Gorman S (2014). Ultraviolet radiation suppresses obesity and symptoms of metabolic syndrome independently of vitamin D in mice fed a high-fat diet. Diabetes.

[6] MacLaughlin J, Holick MF (1985). Aging decreases the capacity of human skin to produce vitamin D3. J Clin Invest.

[7] Pappas LE, Nagy TR (2018). The translation of age-related body composition findings from rodents to humans. Eur J Clin Nutr.

[8] Liu C-Y, Chang C-W, Lee H-C, Chen Y-J, Tsai T-H, Chiau J-SC, Wang T-E, Tsai M-C, Yeung C-Y, Shih S-C (2016). Metabolic damage presents differently in young and early-aged C57Bl/6 mice fed a high-fat diet. Int J Gerontol.

[9] Ng RL, Scott NM, Strickland DH, Gorman S, Grimbaldeston MA, Norval M, Waithman J, Hart PH (2013). Altered immunity and dendritic cell activity in the periphery of mice after long-term engraftment with bone marrow from ultraviolet-irradiated mice. J Immunol.

[10] Lagisz M, Blair H, Kenyon P, Uller T, Raubenheimer D, Nakagawa S (2015). Little appetite for obesity: meta-analysis of the effects of maternal obesogenic diets on offspring food intake and body mass in rodents. Int J Obes (Lond).

[11] Grimpo K, Volker MN, Heppe EN, Braun S, Heverhagen JT, Heldmaier G (2014). Brown adipose tissue dynamics in wild-type and UCP1-knockout mice: in vivo insights with magnetic resonance. J Lipid Res.

[12] Kleiner DE, Brunt EM, Van Natta M, Behling C, Contos MJ, Cummings OW, Ferrell LD, Liu YC, Torbenson MS, Unalp-Arida A (2005). Design and validation of a histological scoring system for nonalcoholic fatty liver disease. Hepatology.

[13] Gorman S, Judge MA, Hart PH (2010). Topical 1,25-dihydroxyvitamin D3 subverts the priming ability of draining lymph node dendritic cells. Immunology.

[14] Gorman S, Tan JW, Yerkovich ST, Finlay-Jones JJ, Hart PH (2007). CD4 + T cells in lymph nodes of UVB-irradiated mice suppress immune responses to new antigens both in vitro and in vivo. J Invest Dermatol.

[15] Black PN, Ahowesso C, Montefusco D, Saini N, DiRusso CC (2016). Fatty acid transport proteins: targeting FATP2 as a gatekeeper involved in the transport of exogenous fatty acids. Medchemcomm.

[16] Angeles TS, Hudkins RL (2016). Recent advances in targeting the fatty acid biosynthetic pathway using fatty acid synthase inhibitors. Expert Opin Drug Discov.

[17] Dorn C, Riener MO, Kirovski G, Saugspier M, Steib K, Weiss TS, Gabele E, Kristiansen G, Hartmann A, Hellerbrand C (2010). Expression of fatty acid synthase in nonalcoholic fatty liver disease. Int J Clin Exp Pathol.

[18] Braunersreuther V, Viviani GL, Mach F, Montecucco F (2012). Role of cytokines and chemokines in non-alcoholic fatty liver disease. World J Gastroenterol.

[19] Nakano T, Cheng YF, Lai CY, Hsu LW, Chang YC, Deng JY, Huang YZ, Honda H, Chen KD, Wang CC (2011). Impact of artificial sunlight therapy on the progress of non-alcoholic fatty liver disease in rats. J Hepatol.

[20] Kim IH, Xu J, Liu X, Koyama Y, Ma HY, Diggle K, You YH, Schilling JM, Jeste D, Sharma K (2016). Aging increases the susceptibility of hepatic inflammation, liver fibrosis and aging in response to high-fat diet in mice. Age (Dordr).

