# Supplementary material for: Regular exposure to non-burning ultraviolet radiation reduces signs of non-alcoholic fatty liver disease in mature adult mice fed a high fat diet: results of a pilot study
Source: BMC Res Notes. 2019 Feb 11;12:78. doi: 10.1186/s13104-019-4112-8 (PMC6371430; doi:10.1186/s13104-019-4112-8)
Supplement: Supplementary file 1 — Additional file 1: Table S1. Ingredient list of low fat diet (LFD) and high fat diet (HFD) fed to mice. Table S2. Power analysis to determine sample sizes of treatment groups to investigate the effects of exposure to low dose UVR on metabolic outcomes in mature ‘older’ mice fed a high fat diet. [file 13104_2019_4112_MOESM1_ESM.docx]

**BMC Research Notes Submission – Supplementary Data**

**Title:** *Regular exposure to non-burning ultraviolet radiation reduces signs of non-alcoholic fatty liver disease in mature adult mice fed a high fat diet: results of a pilot study*

**Authors:** Samantha Teng^1^, Lipi Chakravorty^1^, Naomi Fleury^1^, and Shelley Gorman^1*^

**Additional Table S1** – Ingredient list of low fat diet (LFD) and high fat diet (HFD) fed to mice

| **Ingredients** | **LFD** (SF12-029) | **HFD** (SF12-031) |
| --- | --- | --- |
|  | g/100g | g/100g |
| Sucrose | 10.0 | 10.0 |
| Casein (acid) | 20.0 | 20.0 |
| Canola oil | 5.0 | 2.9 |
| Lard | 0.0 | 20.7 |
| Cellulose | 5.0 | 5.0 |
| Wheat starch | 36.0 | 17.4 |
| Dextrinised starch | 13.2 | 13.2 |
| DL-methionine | 0.3 | 0.3 |
| AIN93_trace minerals | 0.14 | 0.14 |
| Lime (calcium carbonate) | 2.5 | 2.5 |
| Salt (fine sodium chloride) | 0.26 | 0.26 |
| Potassium dihydrogen phosphate | 0.76 | 0.76 |
| Potassium sulphate | 0.16 | 0.16 |
| Potassium citrate | 0.15 | 0.15 |
| Magnesium oxide | 0.17 | 0.17 |
| Dicalcium phosphate | 5.1 | 5.1 |
| AIN93_Vitamins (No vitamin D)^1^ | 1.0 | 1.0 |
| Choline chloride 75% w/w | 0.25 | 0.25 |
| Food colour | 0.02 | 0.02 |
| *% Digestible energy - lipid* | *12.0%* | *45.9%* |
| *% Digestible energy - protein* | *22.0%* | *17.7%* |

^1^American Institute of Nutrition (AIN) trace minerals number 93 is a nutritional supplement used in rodent food designed to promote maintenance of health in adult rodents[22], to which vitamin D was not included.

**Additional Table S2** – Power analysis to determine sample sizes of treatment groups to investigate the effects of exposure to low dose UVR on metabolic outcomes in mature ‘older’ mice fed a high fat diet.

| **Outcome** | **Mock**  (mean±SD) | **UVR**  (mean±SD) | **n(exp)** /treatment | **Actual power^1^** |
| --- | --- | --- | --- | --- |
| Body weight gain (%) | 134.6±4.3, n=7 | 130.8±4.7, n=6 | 23 | 0.81 |
| iBAT weight (g) | 0.31±0.03, n=4 | 0.27±0.06, n=3 | 24 | 0.81 |
| gWAT weight (g) | 1.93±0.38, n=4 | 1.74±0.58, n=3 | 99 | 0.80 |
| Liver histopathology score (x/10) | 6.18±0.57, n=4 | 4.87±0.61, n=3 | 5 | 0.87 |
| AST (U/L) | 79.8±29.7, n=4 | 74.0±8.70, n=3 | 229 | 0.80 |
| Inflammatory foci (#/field) | 0.73±0.23, n=4 | 0.39±0.29, n=3 | 11 | 0.83 |
| *Tnf* mRNA – older mice (*Tnf/Eef1*α*)* | 1.8±1.4x10^-5^, n=4 | 7.1±5.0x10^-6^, n=3 | 15 | 0.82 |
| *Tnf* mRNA – younger mice (*Tnf/Eef1*α*)* | 5.9±11.0x10^-5^, n=14 | 1.2±2.6x10^-5^, n=15 | 47 | 0.81 |

^1^The G*Power (version 3.1.3 for MAC OS10) program was used to predict the sample size needed to reproduce these findings in statistically powered experiments, using a two-tailed student’s *t* test (difference between two independent means, a priori analysis at a power of (1-ß error) probability of 0.8, at an α error probability of 0.05). n(exp) = the number of mice required for sufficient power to observe a significant difference in any subsequent experiment; Mock = mice ‘mock’ irradiated twice a week for 12 weeks; UVR = mice exposed to low dose UVR twice a week for 12 weeks.
